# On the origin of the large hydrophobic solvation driving forces at metal- and oxide-water interfaces†

**DOI:** 10.1039/d5sc03005f

**Published:** 2025-06-13

**Authors:** Mohammed Bin Jassar, Simone Pezzotti

**Affiliations:** a Laboratoire CPCV, Département de Chimie, École normale supérieure, PSL University, Sorbonne Université, CNRS Paris 75005 France mohammed.bin-jassar@ens.psl.eu simone.pezzotti@ens.psl.eu

## Abstract

Recent studies discovered the existence of large hydrophobic solvation driving forces at macroscopically hydrophilic metal/and oxide/aqueous interfaces, which dictate several physical and chemical processes. They arise from the coexistence of local hydrophobic and hydrophilic behaviors. Rationally tuning these newly discovered driving forces by small adjustments of surface (or electrolyte) properties will open exciting perspectives in heterogeneous catalysis, geochemistry, nanofluidics, and electrochemistry. Here, we provide a molecular understanding of the origin of these driving forces, including how they are tuned by surface properties and why they can manifest at very diverse interfaces, from metals to oxides, from conductors to insulators. Our work builds a foundation to control these driving forces for improving existing technologies.

## Abstract 2

This work builds a foundation to control the recently discovered large solvation driving forces at metals and oxides, by tuning the amphiphilic behavior of the interface.

## Graphical abstract

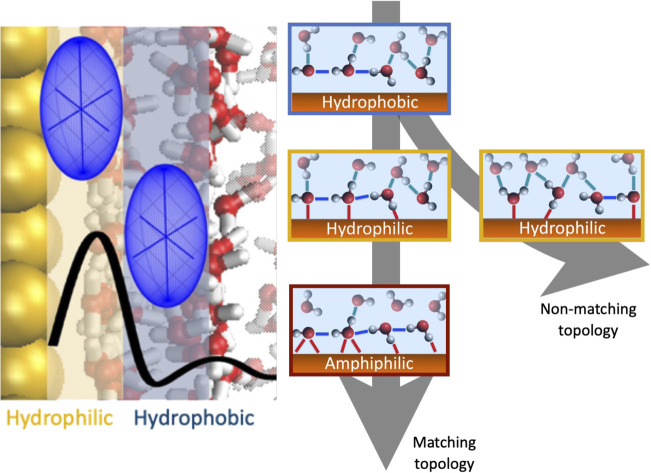

## 1. Introduction

Many physical and chemical processes on our planet and in industry occur at aqueous interfaces, where liquid water meets a solid surface.^1–3^ The termination and reorganization of the water H-bond network in contact with a surface profoundly influence properties such as wetting,^4–8^ surface speciation/hydroxylation,^2,9^ adsorption and transport of ions and molecules,^10–14^ energy transfer,^15–18^ and the outcome of many chemical reactions.^1–3,19,20^ Decades of intensive research traced the landscape of aqueous interfaces. With increasing surface–water interaction strength, a transition from hydrophobic to hydrophilic interfaces is observed, which is subtly modulated by context-dependent parameters such as surface topology and morphology.^4,7,21–29^ Such transition is marked by changes in contact angle at the macroscopic scale and local water density fluctuations (as measured by cavitation free energy) at the molecular scale, the two being quantitatively related.^5^ Water density fluctuations are enhanced at hydrophobic interfaces (high contact angle), while suppressed at hydrophilic interfaces (low contact angle). The hydrophobic–hydrophilic transition is accompanied by a drastic change in the above-mentioned interfacial properties, which influence an enormous variety of fields.^2,3,14,20,21,26,28,30–33^

In the last decade, a growing number of studies reported interfaces that do not fit into the established picture: they exhibit an atypical behavior with mixed properties of both hydrophilic and hydrophobic interfaces.^8,11,13,34–39^ Fascinating wetting and solvation properties are being dug out for many of these interfaces, with promising applications in heterogeneous catalysis, geochemistry, prebiotic chemistry, and electrochemistry, to cite a few.^8,11–13,32,34,37,39–41^ Intriguingly, these interfaces have apparently little in common with each other – they range from geochemical to electrochemical, from metals to oxides, from conductors to insulators.

The first report was on talc surfaces.^34^ There, individual water molecules adsorb strongly (hydrophilic behavior) at low relative humidity due to adhesive surface–water interactions; however, at saturation, a droplet of water beads on the surface, as typical for hydrophobic interfaces. Rotenberg *et al.*^34^ rationalized this duality in terms of a competition between adhesion and cohesion (water–water interactions). Surprisingly, they noted that a water droplet forms on top of a strongly adsorbed water monolayer (adlayer), even on the most adhesive surfaces. For rutile, molecular dynamic (MD) simulations, contact angle measurements, and sum frequency generation (SFG) spectroscopy by Qu *et al.*^35^ proved the surface is strongly wet by a water bilayer. However, the addition of more water results in the formation of a droplet with a finite contact angle on top of it, which led the authors to question whether the interface is hydrophilic or hydrophobic. Experimental measurements of water adsorption enthalpy on alpha-(0001)-quartz surfaces reported strongly exothermic adsorption of a 1st water monolayer, as typical of very hydrophilic surfaces.^36^ This is due to strong H-bonding between quartz SiOH terminations and water, as confirmed by several MD and SFG studies.^2^ However, the addition of a 2nd water layer was surprisingly found much less exothermic and to leave the 1st monolayer structure virtually unperturbed.

At Pt/water interfaces, Limmer, Willard *et al.*^8,37^ initiated a quest on how hydrophobic properties – such as enhanced density fluctuations – arise despite strong metal–water interactions, due to the ordering of the water adlayer on top of the metal. We could later show^11,42^ for Au/water interfaces that strong metal–water interactions template a very ordered water adlayer, where intra-layer water–water H-bonds and interactions with the surface are both maximized. This leaves few spots available for H-bonding between the adlayer and the adjacent water layer. Hence, next to the hydrophilic metal–adlayer interface, where density fluctuations are suppressed, a water–water interface with a low H-bond density is formed, where water density fluctuations are enhanced, as typical of hydrophobic interfaces.

The dual local hydrophobic/hydrophilic nature was recognized to dictate many properties of metal/aqueous interfaces. For example, hydrophobic solvation at the water–water side of the interface regulates adsorption and transport of reactive species across the interface, *e.g.*, by promoting the adsorption of hydrophobic and amphiphilic molecules,^8,11,42^ as well as some ions, influencing the properties of the electric double layer.^12,37,43^ The local solvation environment in this region was further recognized to modulate acid-base chemistry, providing shifts in p*K*_a_ values similar to that observed at hydrophobic interfaces.^32^ The difference in cavitation free energies between the metal–adlayer and water–water interfacial regions was shown to provide large solvation driving forces, up to 0.5 eV, which can modulate the outcome of several chemical reactions in the field of renewable energies.^39,40,44^

Most recently, Gäding *et al.*^13^ discussed how the degree of ordering within the adlayer also dictates friction and osmotic transport at the interface, by determining the corrugation of the free energy landscape of water on the surface. Independently, Li *et al.*^41^ proposed that the low H-bond density at the water–water interface close to the metal limits proton transport across the interface and dominates the kinetic pH effect in hydrogen electrocatalysis.^41,45^

The rising challenge is how these newly identified driving forces can be tuned by adjusting the properties of the metal electrode (or the electrolyte), to improve electrochemical processes. Moreover, the manifestation of apparently similar properties at other aqueous interfaces, including oxides and insulators, suggests that the same driving forces may play key roles in other fields, as, *e.g.*, recently proposed for geochemical and prebiotic processes.^13,39^ Exploiting these emerging opportunities requires molecular understanding.

Here, we aim to fill this gap of knowledge by means of extended MD simulations. We start from a well-characterized electrified Au/water interface, where surface polarization and applied voltage are explicitly treated with the classical constant-potential method,^46^ providing a realistic description of the structural, solvation and charging properties.^11–13,32,40,47^ By taking advantage of the tunability of the theoretical model, we systematically vary the surface properties one-by-one, to explore the effects of surface adhesion, metallicity, applied voltage and topology. This allows us to rationalize why similar mixed hydrophobic/hydrophilic behaviors manifest at very diverse interfaces, as we further confirm with additional DFT-MD simulations of oxide/water interfaces. We hence propose a picture that unites all these diverse interfaces and observations within a single family of systems. We name this family “amphiphilic interfaces”, whose common trait is – in analogy to amphiphilic solutes – to display both a hydrophilic and a hydrophobic side. We identify the molecular origin of amphiphilic behavior, and we rationalize how it is regulated by surface adhesion and topology. These results provide a recipe for fine-tuning hydrophobic solvation driving forces by adjusting surface properties, paving the way toward exploiting them for improving electrochemical and renewable energy processes.

## 2. Results and discussions

### 2.1 Which surface properties unlock the amphiphilic behavior?

We employed constant potential MD^46^ to simulate a series of interfaces derived from an Au(100)/water system by changing the properties of the “Au” electrode. An Au(100) electrode is chosen as the starting point because, despite being reported for a great diversity of systems, amphiphilic behaviors have been extensively characterized at Au(100).^11,13,38,40,42^ The adopted classical constant-potential method allows us to explicitly model the electronic response of the electrode to the applied voltage by means of fluctuating atomic charges on the surface Au atoms. The electrode atomic charges are represented with atom-centered Gaussian charge distributions, whose magnitude fluctuates in response to changes in the electrolyte structure while obeying the constant potentital constraint (determined by the applied voltage).^46^ Instead, the width of the Gaussian distribution (*ζ*) is fixed and is a property of the electrode material: changing the width is an effective way to tune the electrode metallicity, *i.e.* its conductor/insulator character.^47^ This method has been extensively tested and provides a realistic description of structural and solvation properties of electrified Au/water interfaces,^11,32,38,40,46^ as also confirmed by comparison to most recent DFT-level machine learning potential MD.^13^ It also provides a good description of the electronic properties, *i.e.*, the differential capacitance, in agreement with the latest DFT-MD and experimental studies.^47^ With this theoretical setup, we can explore the effect of distinct surface properties on the amphiphilic behavior in a systematic way, by tuning one-by-one the parameters of the constant potential electrode and that for the interaction potential between the electrode and water.

The surface properties suspected to be relevant are: (i) the strength of adhesive surface–water interactions, since amphiphilic behavior was observed for strongly adhesive surfaces; (ii) surface metallicity, as amphiphilicity was intriguingly observed for both insulators and conductors; (iii) applied voltage, as many amphiphilic interfaces are electrified. Adhesive surface–water interactions were tuned by the *ε* parameter in the Lennard-Jones potential between Au and water (O-atom). Metallicity was tuned by changing the image plane position (which corresponds to changing the width *ζ* of the Gaussian charge distribution used to represent the atomic charges in Au,^47^ see Methods). The image plane *z*_im_ defines the effective location of the induced screening charge in response to an external perturbation, such as applied electric field or surface charge. When the image plane position *z*_im_ − *z*_a_ is positive, it lies outside the material, indicating that the screening charge is localized near the interface. This behavior is characteristic of good metallic screening. In contrast, a negative image plane position places it inside the material, implying that the screening charge is more delocalized and distributed within the bulk, which is indicative of weaker or non-metallic screening behavior. The applied voltage was varied within the water electrochemical window, from the point of zero charge (PZC = 0 V) to −0.5 V on the negative side and to +0.5 V on the positive side.

The influence of these surface properties is evaluated by analysis of water density fluctuations (as quantified by cavitation free energies), which provide a well-established measure of local hydrophobicity:^4,5,8,11,21,48,49^ the free energy cost to spontaneously form a small cavity in the liquid is reduced compared to bulk water at hydrophobic interfaces (δ*μ*_cavity_ < 0) due to enhanced fluctuations of the water density, while δ*μ*_cavity_ ≥ 0 at hydrophilic interfaces. δ*μ*_cavity_(*z*) profiles along the *z* distance from the surface (where δ identifies a difference between the *z*-position at the interface and the bulk) were computed from the MD simulations (see Methods). Corresponding contact angle values were deduced with the approach of ref. 5 and are shown in the ESI.†

**Fig. 1:**
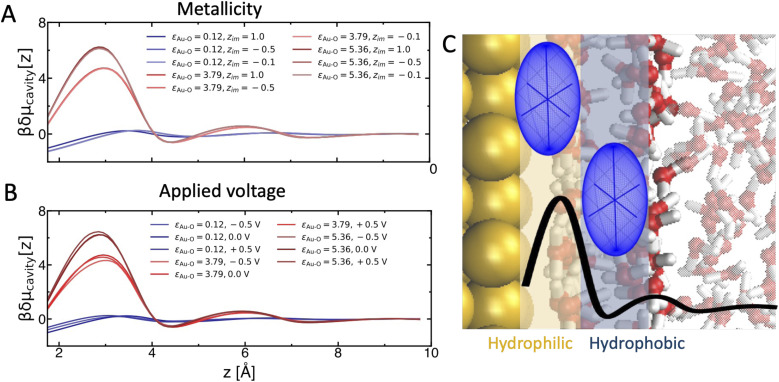
Mapping amphiphilic interfaces as a function of surface properties. (A) and (B) compare the effect of surface–water interaction strength (*ε*_Au−O_ in kJ mol^−1^) *vs.* surface metallicity (*z*_im_ in Å) and applied voltage (Δ*V*), respectively. For comparison, *ε*_Au−O_ = 3.79 kJ mol^−1^ and *z*_im_ = 1.0 Å for the original Au(001) surface (see Methods). Amphiphilic behavior is quantified by cavitation free energy (δ*μ*_cavity_, in units of *β* = 1/*k*_B_*T*) profiles along the z distance from the surface. δ*μ*_cavity_(*z*) monotonically decreases when approaching a hydrophobic surface, as in the profiles with *ε*_Au−O_ = 0.12 kJ mol^−1^ (blue curves). Amphiphilic behavior is observed for the two high *ε*_Au−O_ values, independently of metallicity and voltage. (C) Illustration of the amphiphilic behavior at Au/water (red oxygen and white hydrogen). The black curve shows the typical δ*μ*_cavity_(*z*) profile (for the illustrated ellipsoidal cavity (with volume of 2.00 Å × 2.00 Å × 1.75 Å), with exceptionally high δ*μ*_cavity_(*z*) at the hydrophilic surface-adlayer interface, and a minimum at the adjacent water–water interface. Such exceptionally large variations within a few angstroms from the surface are at the origin of the recently discovered hydrophobic solvation driving forces at metal- and oxide-water interfaces.

Fig. 1A and B compare the effect of adhesive interactions *vs.* metallicity and applied voltage, respectively. Strikingly, adhesion emerges as the dominant parameter, while comparably smaller modulations of δ*μ*_cavity_(*z*) arise from the surface being insulator/conductor or neutral/charged. The adhesive forces were controlled by tuning the interaction strength between the Au electrode and the oxygen atom of water, *i.e.*, the *ε*_Au–O_ parameter in the adopted Lennard-Jones potential. Three values of *ε*_Au–O_ were chosen to represent different surface interaction regimes: (i) the original Au electrode value from ref. 50, *ε*_Au−O_ = 3.79 kJ mol^−1^ (see Methods for more details about this choice); (ii) a weak interaction case, *ε*_Au−O_ = 0.12 kJ mol^−1^, simulating a hydrophobic surface; (iii) a stronger interaction case, *ε*_Au−O_= 5.36 kJ mol^−1^. At low interaction strength (*ε*_Au−O_ = 0.12 kJ mol^−1^), we systematically recover the typical δ*μ*_cavity_(*z*) profile of a hydrophobic interface: δ*μ*_cavity_(*z*) monotonically decreases when approaching the surface.^21,48^ Instead, for interaction strength equal (*ε*_Au−O_ = 3.79 kJ mol^−1^) or higher (*ε*_Au−O_ = 5.36 kJ mol^−1^) than for the original Au(100) surface, the amphiphilic behavior arises, independently of metallicity and applied voltage. As illustrated in Fig. 1C, this is characterized by exceptionally high δ*μ*_cavity_(*z*) at the hydrophilic interface formed between the surface and the water adlayer (*z* < 3.5 Å), where there is a high density of water–surface interactions and intra-adlayer water–water H-bonds.^42^ Instead, δ*μ*_cavity_(*z*) becomes negative (*i.e.*, hydrophobic-like) at the adjacent water–water interface, where the density of inter-layer H-bonds between the adlayer and the adjacent water layer is low. Such exceptionally large structure and cavitation free energy variations within a few angstroms from the surface are at the origin of the many hydrophobic solvation driving forces discovered in recent studies at amphiphilic interfaces.^8,11–13,32,34,37,39–41,44,45^ From the results of Fig. 1, we can already rationalize why these driving forces were observed for both insulators and conductors, electrified and neutral surfaces: they can manifest with sufficiently strong surface–water interactions, independently of the nature of the surface.

**Fig. 2:**
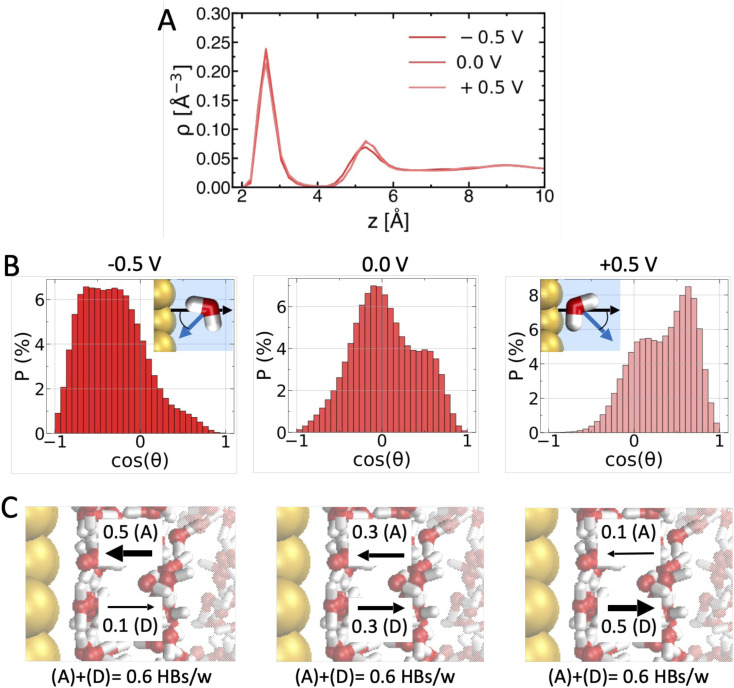
Why are Δ*V* variations within the water electrochemical window insufficient to induce/prevent amphiphilic behavior. (A) Δ*V* induced changes in water oxygen density profiles (for *ε*_Au−O_ = 3.79 kJ mol^−1^ and *z*_im_ = 1.0 Å). (B) Water dipole orientation (cos *θ*, see scheme) distributions for different Δ*V*. (D) Accompanying changes in the number of H-bonds per water molecule (HBs/w) formed between the adlayer and the adjacent layer: adlayer water mostly receives HBs (0.5 *vs.* 0.1 HBs/w) from water in the next layer at Δ*V* = −0.5 V, while it mostly donates HBs at Δ*V* = +0.5 V. However, the total density of HBs/w – that dictates δ*μ*_cavity_(*z*) – remains constant (0.6 HBs/w). Error bars on HBs/w values are <± 0.05 HBs/w.

This may appear surprising at first glance, since both metallicity and applied voltage influence the way water molecules interact with the surface and with themselves.^11,47,51^ To understand this, we characterize the effect of applied voltage on the interfacial water H-bond network in Fig. 2. Tuning the voltage across the PZC does not substantially influence the water oxygen density profile (Fig. 2A), in particular the height and position of the first density peak corresponding to the water adlayer. However, it strongly alters the orientation of water molecules within the adlayer (Fig. 2B): water molecules are mostly oriented with their dipole parallel to the surface at PZC = 0 V (probability maximum for cos *θ* ≃ 0), but reorient pointing their dipole toward the negative surface (cos *θ* < 0 for −0.5 V) and away from the positive surface (cos *θ* > 0 for +0.5 V). As shown in Fig. 2C, the reorientation alters inter-layer H-bonding at the water–water interface: adlayer water mostly accepts H-bonds from water in the subsequent layer at negative voltage, while it mostly donates at positive voltage. However, the total density of H-bonds remains constant. Since what matters for density fluctuations is the density of H-bonds that has to be perturbed to create a cavity, δ*μ*_cavity_(*z*) is not very sensitive to applied voltage variations. A similar molecular rationalization applies for the effect of metallicity, too (Fig. S1 in the ESI†).

### 2.2 Locating the crossover from hydrophobic to hydrophilic to amphiphilic

**Fig. 3:**
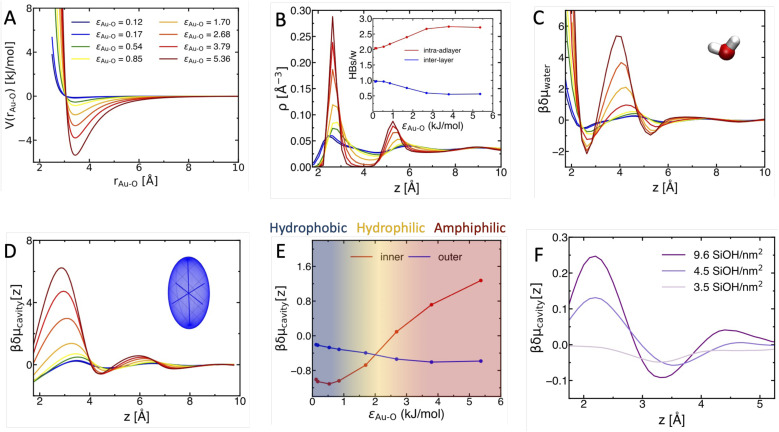
The hydrophobic-hydrophilic-amphiphilic crossover with increasing surface–water interactions, *ε*_Au−O_. (A) Lennard-Jones potential *V*(*r*_Au−O_) for each *ε*_Au−O_. (B) Water oxygen density profiles, showing the progressive appearence of an ordered water adlayer (1st density peak). (Inset) accompanying changes in the number of water–water H-bonds (HBs/w) formed within the adlayer (red) and between the adlayer and the adjacent water layer (blue). (C) Water chemical potential δ*μ*_water_ across the interface, as computed from the density profiles. (D) δ*μ*_cavity_(*z*) (in units of *β* = 1/*k*_B_*T*) profiles (see Fig. S2 in ESI† for the corresponding changes in contact angle, which varies from 90° to full wetting with increasing *ε*_Au−O_). (E) δ*μ*_cavity_(*z*) changes are quantified by plotting the values at *z* = 1.8 Å (inner) and 4.5 Å (outer) as a function of *ε*_Au−O_. The color gradient highlights the progressive crossover: δ*μ*_cavity_(*z*) inner <outer at a hydrophobic interface (blue), inner ≃ outer at a hydrophilic interface (orange), and inner >outer at an amphiphilic interface (brown). (F) The hydrophilic-amphiphilic crossover is also observed for silica–water interfaces, where the adhesive force is increased by increasing surface hydroxylation rate (SiOH/nm^2^) instead of *ε*_Au−O_, showing the generality of our findings.

Leveraging on the gained knowledge, we can anticipate that, if such a crossover exists, it must appear with increasing surface–water interaction. We hence examine in Fig. 3 how the structure of the interface and δ*μ*_cavity_(*z*) change by continuously varying *ε*_Au−O_ (as shown in Fig. 3A with the corresponding Lennard-Jones potentials) from the value of 0.12 kJ mol^−1^, for which we observed hydrophobic behavior, to the value of 5.36 kJ mol^−1^ (amphiphilic behavior). The larger *ε*_Au−O_, the more the water (O-atom) density profile (Fig. 3B) becomes structured at the solid–liquid interface, with the progressive appearance of a 1st density peak at around 2.8 Å, indicative of the formation of a water adlayer, followed by a depleted inter-layer region and by a 2nd density peak. Concomitantly (inset), the number of intra-layer H-bonds formed between adlayer water molecules increases (red curve), while that of inter-layer H-bonds formed by the adlayer with the water molecules in the 2nd layer decreases (blue), until both reach a plateau at *ε*_Au−O_ ≃ 3 kJ mol^−1^. At the plateau, the connectivity within the adlayer is maximized, which corresponds to the fewest amount of spots remaining available for H-bonding with the 2nd layer. From the density profiles, we can quantify the water chemical potential at the interface: δ*μ*_water_(*z*) = −ln *P*_w_(*z*), with *P*_w_(*z*) being the probability to find a water molecule at a given *z*-distance from the surface (normalized by that in the bulk). With increasing *ε*_Au−O_, water adsorption in the adlayer (1st minimum in the δ*μ*_water_(*z*) profiles) becomes more favorable, boosted by the increased water–water connectivity, while the free energy barrier to remove a water molecule from the adlayer (δ*μ*_adlayer_, *i.e.*, the difference between the 1st δ*μ*_water_ minimum and the subsequent maximum) increases. These changes appear smooth and continuous.

Strikingly, they are accompanied by a continuous crossover from δ*μ*_cavity_(*z*) profiles typical of hydrophobic interfaces (blue in Fig. 3D) to δ*μ*_cavity_(*z*) profiles typical of an amphiphilic interface (red/brown). As soon as adhesive interactions start to increase, spatial fluctuations, within ∼8 Å from the surface, become progressively evident in the free energy profiles. This is a well-know consequence of the layering of water close to a hydrophilic surface, as observed in the density profiles. However, from orange to brown curves in Fig. 3D, the magnitude of the δ*μ*_cavity_ spatial fluctuations grows suddenly, beyond the standard layering effect. The metal- and oxide-water interfaces with atypical wetting properties, where a water droplet beads with a finite contact angle on top of a strongly bound water adlayer, systematically exhibit this kind of δ*μ*_cavity_ profiles.^8,11,34,39^ Once again, it is the large magnitude of the spatial fluctuation of δ*μ*_cavity_ (and of H-bonding properties, Fig. 3B) that gives rise to the hydrophobic solvation driving forces at these interfaces that we aim to rationalize and tune.

The transition toward the amphiphilic behavior is marked by the progressive appearance of a δ*μ*_cavity_ maximum at *z* < 3.5 Å (where the cavity is formed inner-sphere, *i.e.*, in direct contact with the surface and within the adlayer) and a minimum at ∼4.5 Å (where the cavity is formed outer-sphere, *i.e.*, at the adjacent water–water interface where it is separated from the surface by the adlayer). These changes are quantified in Fig. 3E by plotting the δ*μ*_cavity_ values at ∼1.8 Å (red, inner-sphere) and at ∼4.5 Å (blue, outer-sphere) as a function of *ε*_Au−O_. At low *ε*_Au−O_, inner-sphere is more favorable than outer-sphere cavity formation, and both are favored with respect to bulk (δ*μ*_cavity_ < 0). This indicates hydrophobic behavior, as δ*μ*_cavity_ monotonically decreases when approaching a hydrophobic surface.^21,48^ With increasing *ε*_Au−O_, the δ*μ*_cavity_ value for inner-sphere increases, approaches zero, and becomes positive, while crossing with the decreasing value for outer-sphere (at around *ε*_Au−O_ = 2 kJ mol^−1^ in our model). In this range, δ*μ*_cavity_ for inner-sphere, outer-sphere and bulk are most similar to each other, which is typical of hydrophilic interfaces.^21,39,48^ This is the canonical hydrophobic–hydrophilic transition. However, when further increasing surface–water interactions, inner- and outer-sphere δ*μ*_cavity_ diverge, and the interface progressively partition into a hydrophilic side (the surface–adlayer interface) and a hydrophobic side (the subsequent water–water interface), giving rise to the amphiphilic behavior.

A progressive hydrophilic–amphiphilic crossover hence takes place when maximizing surface adhesion by increasing the attractive interaction between surface site and water molecule. This is typically the way surface adhesion varies across metal surfaces, *e.g.*, from Au to Cu to Pt.^50,52^ However, this does not apply to oxides, for which amphiphilic behaviors have been reported, too.^34–36,39^ There, the adhesive interactions are primarily modulated by the amount of surface (–OH) terminations that interact strongly with water. A typical example are silica/water interfaces, where surface adhesion increases monotonically with the degree of hydroxilation, *i.e.*, the density of surface SiOH termination that form strong H-bonds with water (which is tuned by changing the way the surface is heat-treated).^28,53^ To explore the generality of our findings, we also performed our analysis on three well-characterized silica surfaces with increasing hydroxylation rate of 3.5 SiOH per nm^2^, 4.5 SiOH per nm^2^ and 9.6 SiOH per nm^2^ (from previously performed DFT-MD simulations^39^). These model systems were shown to reproduce well the properties of heat treated amorphous silica, amorphous silica and α-(0001)-quartz surfaces in contact with liquid water, respectively.^28,39,53^ The corresponding δ*μ*_cavity_(*z*) profiles (Fig. 3F) show again a smooth, continuous crossover from a canonical hydrophilic behavior (3.5 SiOH per nm^2^), with similar δ*μ*_cavity_(*z*) values in inner-, outer-sphere and bulk, to the amphiphilic quartz/water interface (9.6 SiOH per nm^2^), where the hydrophilic and hydrophobic sides (with suppressed and enhanced density fluctuations, respectively) emerge.^39^ We show in Table S1 of the ESI† that the hydrophilic–amphiphilic transition is, also in this case, accompanied by an increasing connectivity within the adlayer and decreasing H-bonding between the adlayer and the second water layer. The hydrophilic–amphiphilic crossover is accessible independently of the way surface adhesion is increased.

### 2.3 Understanding the crossover

**Fig. 4:**
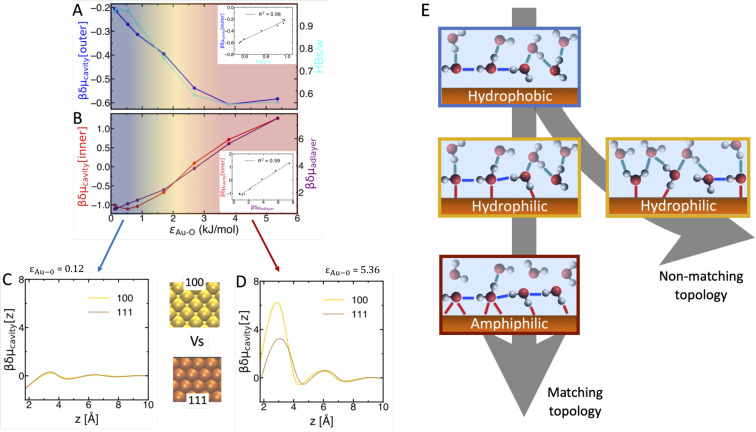
Molecular understanding of the crossover. (A) δ*μ*_cavity_(*z*) changes *vs. ε*_Au−O_ at the hydrophobic water–water side of the interface (from Fig. 3E, outer at *z* ≈ 4.5 Å) are dictated by changes in the number of inter-layer H-bonds (HBs/w, Fig. 3B). The inset highlights the linear correlation between the two. (B) δ*μ*_cavity_(*z*) changes at the hydrophilic side (inner at *z* ≈ 1.8 Å) are dictated by the free energy cost to displace a water molecule from the adlayer (δ*μ*_adlayer_), which depends on both water–surface and water–water interactions. The inset correlates δ*μ*_cavity_ (inner) with δ*μ*_adlayer_. (C) and (D) Effect of surface topology at different sides of the crossover, evaluated by comparing δ*μ*_cavity_(*z*) profiles at Au(100) and Au(111) derived surfaces with same *ε*_Au−O_, *z*_im_ = 1.0 Å, and Δ*V* = 0 V, for *ε*_Au−O_ = 0.12 kJ mol^−1^ (C, hydrophobic), and *ε*_Au−O_ = 5.36 kJ mol^−1^ (D, amphiphilic). (E) Updated understanding of aqueous interfaces, combining our results with recent literature. The arrows follow the crossover with increasing surface adhesion. The sketches illustrate the accompanying changes in surface-water (red lines), intra-adlayer (blue) and inter-layer (cyan) H-bonds. For increasingly adhesive surfaces, the newly discovered amphiphilic behavior can be reached, beyond the canonical hydrophobic–hydrophilic crossover, if the surface topology matches that of the water adlayer.

Fig. 4 shows that such crossover is quantitatively dictated by the surface induced structural changes in the surface–adlayer and water–water sides of the interface. As discussed above, local hydrophobicity, *i.e.* the value of δ*μ*_cavity_(*z*), depends on the number and strength of water–water and surface–water interactions that have to be perturbed to form a small cavity. For a cavity inscribed in the water–water interface, this cost in dictated by the inter-layer water–water H-bonds, which all have similar strength. The number of such H-bonds (HBs/w) is – to a good approximation – linearly proportional to the local δ*μ*_cavity_ (Fig. 4A). Starting from the least adhesive, most hydrophobic surface, increasing *ε*_Au−O_ induces more ordering within the adlayer, with fewer spots remaining available for inter-layer H-bonding with the 2nd water layer, causing both HBs/w and δ*μ*_cavity_ to decrease. Once the adlayer structure is fully ordered and does not change anymore with *ε*_Au−O_ (at >3 kJ mol^−1^ in our model), the number of inter-layer HBs/w reaches a plateau, and so does the local δ*μ*_cavity_.

At the surface-adlayer interface (Fig. 4B), cavity formation requires displacing water molecules from the ordered adlayer structure, which involves perturbing both surface–water and intra-adlayer water–water HBs. Here, δ*μ*_cavity_ is dictated by surface desolvation, *i.e.* by the free energy cost to remove water molecules from the adlayer (δ*μ*_adlayer_).^38,40,42,54^ At low *ε*_Au−O_, in the hydrophobic domain, water–surface interactions are weaker than water–water H-bonds and the adlayer structure is only little sensitive to the *ε*_Au−O_ increase. Thus, both δ*μ*_adlayer_ and local δ*μ*_cavity_ are almost insensitive to changes in surface adhesion, until surface–water overcomes water–water interactions (at *ε*_Au−O_ ≃ 1.5 kJ mol^−1^ in our model). After that, both the strength of water–surface interactions and the number of intra-adlayer HBs/w monotonically increase with *ε*_Au−O_, causing δ*μ*_adlayer_ and δ*μ*_cavity_ to increase. Upon entering the amphiphilic domain, the number of intra-adlayer HBs/w saturates, but δ*μ*_adlayer_ and δ*μ*_cavity_ keep increasing due to the continuous increase in surface adhesion. Therefore, the mechanism of the crossover is the following. For hydrophobic interfaces, water–water interactions dominate and increasing surface adhesion has little effect on wetting properties until surface–water overcomes water–water interactions. In the hydrophilic domain, the more adhesive the surface, the more ordered the water adlayer, the lower the inter-layer H-bond density in the adjacent water–water region. These structural changes progressively cause density fluctuations in the two regions to diverge, with formation of a locally super-hydrophilic surface-adlayer interface followed by a locally hydrophobic water–water interface.

### 2.4 The effect of surface topology

The prerequisite to enter the amphiphilic domain is hence the formation of a horizontally ordered adlayer where both surface–water and water–water interactions are maximized. This requires the pattern on the surface to be commensurate to the water network.^8,13,25,29,31,55^ If not, the formation of surface–water interactions locally perturbs the water network, causing either the breaking or the (out-of-plane) reorientation of intra-adlayer water–water H-bonds, as described in many previous studies.^29,31,55–59^ This prevents the formation of horizontally ordered adlayer structures, leading to canonical hydrophilic interfaces that cannot exhibit amphiphilic behaviors, as typical for most biological and oxide surfaces.^25,56,59^ Hence, amphiphilic behaviors must be strongly modulated by surface topology, *i.e.* by the pattern formed by the surface sites that interact strongly with water. To explore the topology effect, we constructed model interfaces with the same *ε*_Au−O_, metallicity, and applied voltage as for the Au(100) derived surfaces, but starting from a Au(111) surface, instead. Fig. 4C and D compare δ*μ*_cavity_ profiles at Au(100) *vs.* Au(111) model surfaces in the hydrophobic and amphiphilic domains, respectively. Almost identical profiles are observed at low *ε*_Au−O_: surface topology is almost irrelevant for hydrophobic interfaces, in agreement with ref. 59. This is rationalized by considering that water–water overcomes surface–water interactions; therefore, the interfacial water network is dictated by water–water H-bonds instead of adapting to the surface.^31,56,59,60^

The topology effect is remarkable in the amphiphilic domain, instead. The value of δ*μ*_cavity_ at the hydrophilic surface–adlayer interface (*z* < 4 Å) changes by a factor of two between Au(100) and Au(111). This large topology effect is in line with previous studies comparing the wetting of different facets of the same metal.^8,13,38^ As shown by water density and δ*μ*_water_ profiles in Fig. S3 of the ESI,† we find that surface topology dictates the degree of ordering within the adlayer, and as a consequence δ*μ*_adlayer_. This, in turn, dictates the extent to which amphiphilic behaviors manifest. The effect of surface topology varies depending on where an interface is placed along the hydrophobic–hydrophilic–amphiphilic crossover.

## 3. Conclusion

In summary, we propose a framework to rationalize the growing number of diverse interfaces that defy binary hydrophobic or hydrophilic definitions, classifying them within a single family: amphiphilic interfaces. Amphiphilic interfaces are characterized by the coexistence of a locally hydrophilic (surface-adlayer) and hydrophobic (adlayer-2nd layer) side. We identified adhesive surface–water interactions and surface topology as the key factors driving the amphiphilic behavior. As surface–water interaction increases, we traced the continuous transitions from hydrophobic to hydrophilic and then to amphiphilic interfaces using cavitation free energy analysis. As summarized in Fig. 4E, the transition is governed by the surface-induced structural changes in both surface–adlayer and water–water sides of the interface. We show that amphiphilic behaviors can arise for both conductors and insulators, oxides and metals, as long the surface is able to induce a horizontally ordered adlayer structure. This depends on surface topology, as a good geometric match between surface and adlayer patterns is required to maximize surface-adlayer and intra-adlayer H-bonds simultaneously.

The concept of amphiphilic interfaces changes the way we understand hydrophilicity at solid/liquid interfaces in a similar way as the concept of amphiphilic molecules shaped our understanding of hydrophilicity for molecules. At the macroscopic scale, a molecule's hydrophilicity is determined by its solubility. However, at the molecular level, many water-soluble molecules, such as alcohols or amino acids, are composed of a hydrophilic and a hydrophobic side, which confers them their unique chemical and physical properties. In the same way, interfaces classified as macroscopically hydrophilic by contact angle measurements can exhibit, at the molecular level, both a hydrophobic and a hydrophilic side, which leads to the large hydrophobic solvation driving forces that keep being dug out in recent years.^8,11–13,32,34,37,39–41,44,45^ These include the low H-bond density at the hydrophobic side of metal/water interfaces, which is suspected to determine the free energy barrier for proton transport and hydrogen evolution reaction in electrochemistry,^41^ the degree of ordering within the adlayer, which shapes the surface free energy landscape of water that regulates friction and osmotic flow in nanofluidics,^13^ the difference in solvation free energies between hydrophobic and hydrophilic sides that modulate reaction free energies for heterogeneous catalysis (*e.g.*, for CO_2_ reduction for renewable energies) and prebiotic chemistry,^32,39,40^ and many more. The present work provides a unified ground to rationalize all these driving forces and predict how they can be controlled by adjusting the interface composition. This may open exciting perspectives in interface science. For instance, in future studies, the approach outlined here can be expanded to study reactive, functionalized, or defective surfaces, by evaluating water density fluctuations (or corresponding structural-based descriptors for higher spatial/temporal resolution^29^) before and after the surface modification. This will help understanding how the uncovered solvation driving forces can be controlled in practical applications by playing with the composition of the surface.

## 4. Methods

All simulations were performed using the constant potential classical MD code MetalWalls,^46^ on a 3.66 × 3.66 × 7.00 nm box containing 2381 SPC/E^61^ water molecules confined between two identical planar Au electrodes of five atomic layers. 2D periodic boundary conditions were applied on *x* and *y*. For Au, Lennard-Jones parameters from Heinz *et al.*^50^ were used, in combination with Lorentz–Berthelot mixing rules. For each simulation, the system was first equilibrated for 500 ps in the NVT ensemble at *T* = 298 K with a 1 fs timestep, followed by 500 ps, where the electrodes acted as pistons to maintain constant pressure, until the system's density had converged. Then, NVT production runs were propagated with a 2 fs timestep for at least 30 ns. For all NVT simulations, a Nosé–Hoover thermostat^62^ chain was used with a time constant of 1 ps. The choice of interaction potential and metallicity for the original Au electrode was shown in previous studies^12,13,47,50^ to reproduce reasonably well structural, spectroscopic properties, differential capacitance, and cavitation free energies compared to available experiments and *ab initio*-level simulation results. However, it does not provide quantitative agreement with *ab initio* studies, *e.g.*, in terms of the location of adlayer water molecules, *i.e.*, hollow site *vs.* top site,^63^ and water density profile.^64^ Therefore, our results and discussions focus on trends as a function of surface properties.

We adopted the approach of ref. 47 to tune surface metallicity by the width of the atom-centered Gaussian charge distributions (*ζ*) on the topmost electrode atoms as a function of the position *r*:1
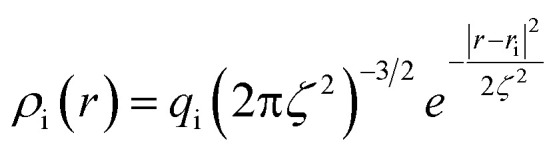
where *ρ*_i_(*r*) and *r*_i_ are the charge distribution and the position of the electrode atom *i*, respectively, while *q* is the atomic charge. The corresponding change in image plane is hence obtained from the response charge density, *δ_ρ_*(*z*)|*_D_* = *ρ_D_*(*z*) – *ρ*_*D*=0_(*z*):^65^2
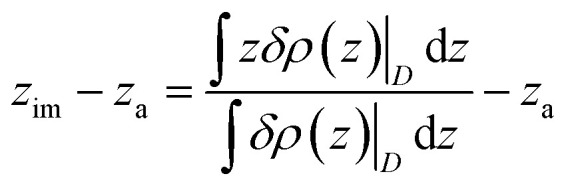
where *z*_a_ denotes the position of the topmost layer of the Au electrode (in our case, *z*_a_ = 0).

Cavitation free energy profiles were obtained by computing the probability *P*_*v*,*s*_(0,*z*) of finding zero water oxygen atoms within a defined probing volume *v* (with shape *s*) at varying distances z from the surface. The probability *P*_*v*,*s*_(0,*z*) is obtained from MD simulations by analyzing the statistics of fluctuations in waterdensity. It is quantitatively related to the free energy cost of cavity formation (Δ*μ_v,s_*) by:^8,11^

3 *P*_*v*,*s*_(0,*z*) = *e*^−*β*Δ*μ*_*v*,*s*_(*z*)^

where *β* = 1/*k*_B_*T*. The difference in cavitation free energy between the *z*-position at the interface and the bulk, denoted δ*μ*_cavity_(*z*) in the profiles of Fig. 1, 3 and 4, is hence given by:

4 δ*μ*_cavity_(*z*) = Δ*μ*^int^_*v,s*_(*z*) − Δ*μ*^bulk^_*v,s*_

We systematically employed an ellipsoidal probing volume of 2.00 × 2.00 × 1.75 Å, which is small enough to fit within the hydrophilic (and hydrophobic) side of an amphiphilic interface. This is essential to avoid mixing cavitation free energy contributions from the two sides, as detailed in ref. 42. H-bond analysis adopted the Luzar-Chandler distance + angle criterion.^66^

## Author contributions

S. P. conceived the project, M. B. J. performed the research. Both authors edited the manuscript.

## Conflicts of interest

There are no conflicts to declare.

## Supplementary Material

SC-OLF-D5SC03005F-s001

## Data Availability

The input files, initial and representative configurations for all the performed MD simulations, as well as the raw computational data for all figures have been deposited in Zenodo (https://zenodo.org/records/14810689).
