# Supplementary material for: On the origin of the large hydrophobic solvation driving forces at metal- and oxide-water interfaces
Source: Chem Sci. 2025 Jun 13;16(28):12823–32. doi: 10.1039/d5sc03005f (PMC12224402; doi:10.1039/d5sc03005f)
Supplement: SC-016-D5SC03005F-s001 [file SC-016-D5SC03005F-s001.pdf]

Supplementary Information (SI) for Chemical Science.
This journal is © The Royal Society of Chemistry 2025

Supporting information for: On the origin of the large hydrophobic solvation driving forces at metal- and oxide-water interfaces

Mohammed Bin Jassar* and Simone Pezzotti*

Laboratoire CPCV, Département de Chimie, École Normale Supérieure, PSL University, Sorbonne University, CNRS, 75005 Paris, France

E-mail: mohammed.bin-jassar@ens.psl.eu; simone.pezzotti@ens.psl.eu

Additional Methodological Details

The input files, initial and representative configurations for all the performed MD simulations, as well as the raw computational data for all figures have been deposited in Zenodo (https://zenodo.org/records/14810689).

The Au (111) surface was constructed using the CHARMM software package,[1,2] with a box dimension of 3.75×4.00×7.02 nm, containing 2,381 SPC/E[3] water molecules confined between two identical planar Au electrodes. Similar to the Au (100), all simulations for the Au (111) were performed using the constant potential classical MD code MetalWalls.[4]

For the three silica-water interfaces, Born–Oppenheimer DFT-MD simulations were carried using CP2K/Quickstep[5] with the PBE–D3 functional,[6,7] GTH pseudopotentials,[8] and a hybrid basis set consisting of plane waves (cutoff of 400 Ry) and DZVP-MOLOPT-SR. The simulation cell dimensions were 9.8 Å × 8.5 Å × 32 Å for 9.6 SiOH/nm^2-water, 13.4 Å × 13.3 Å × 37 Å for 4.5 SiOH/nm^2-water, and 12.67 Å × 13.27 Å × 37 Å for 3.5 SiOH/nm^2-water, consistent with previous studies 9–12, which utilized amorphous slab models from ref. 13. The simulations were conducted in the NVT ensemble at 300 K using a Nosé–Hoover thermostat[14] (chain length = 3, time constant = 100 fs), running for a minimum of 160 ps following 10 ps of equilibration.

The calculation of the contact angle θ was performed based on the criteria in ref.[15] where it is related to the cavitation free energy through Eq.1

$$\delta\mu_{\text{cavity}}(z^*) = m\left[\cos(\theta) - \cos(\theta_0)\right], \tag{1}$$

where z* represents the position of the first minima in the cavitation free energy profile, $m = \Delta\mu_{v,s}^{bulk}\ /2(1 + \cos(45°))$, and $\theta_0 = 45°$ represents the boundary between hydrophobic and hydrophilic interactions.

Additional Analysis

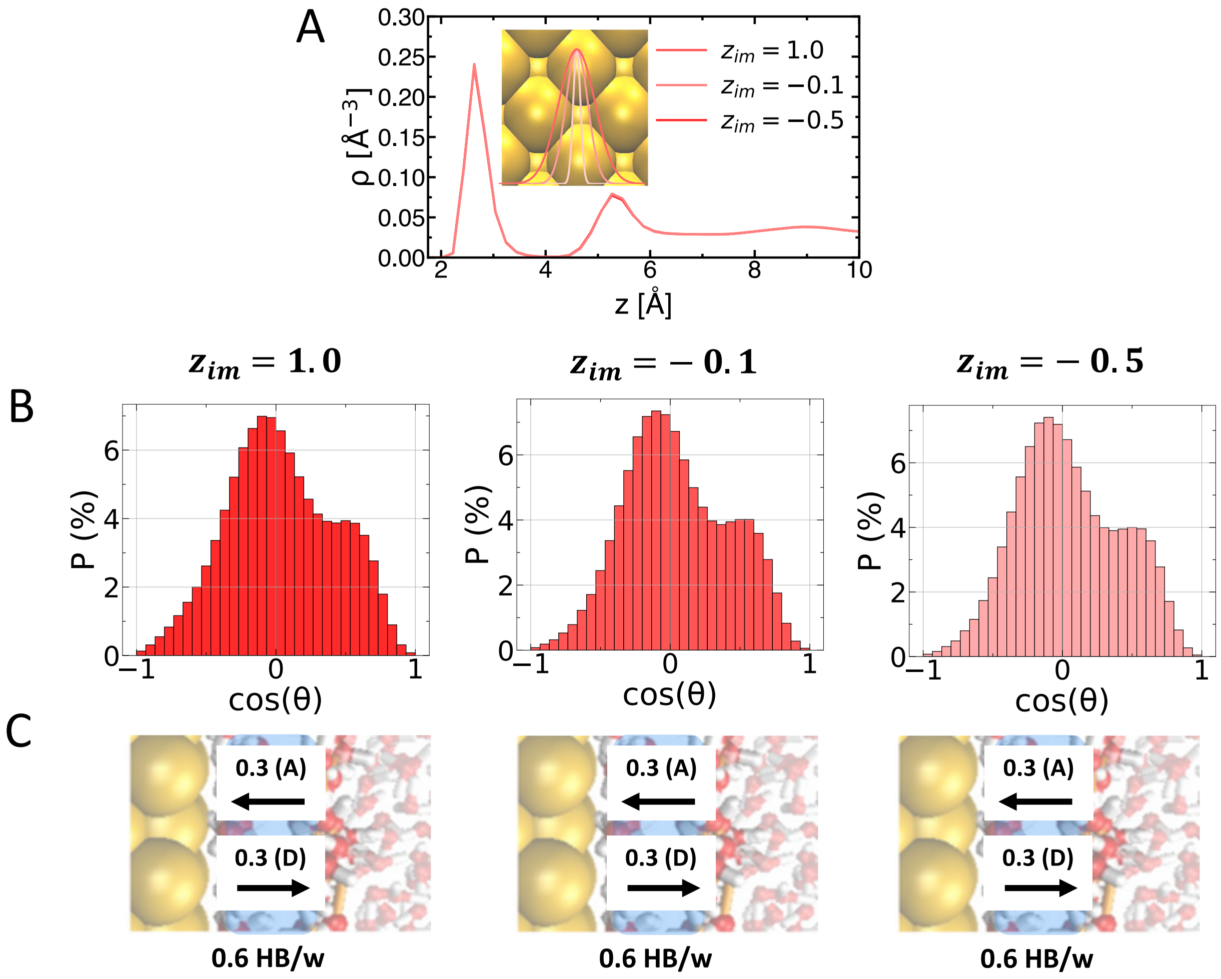

Figure S1: Why are z_{im} (in Å) variations insufficient to induce or prevent amphiphilic behavior? (A) ζ-induced changes (represented by the corresponding z_{im}) in water oxygen density profiles (for $\epsilon_{Au-O} = 3.79\,\mathrm{kJ/mol}$ and $\Delta V = 0V$). (B) Representation of the width of the atom-centered Gaussian distribution over Au, which is varied to tune surface metallicity (smaller ζ results in a wider Gaussian). (C) Water dipole orientation distributions for different z_{im}. (D) Accompanying changes in the number of inter-layer hydrogen bonds per water molecule (HBs/w) formed between the adlayer and the adjacent water layer: the total density of inter-layer HBs/w—which dictates $\delta\mu_{\mathrm{cavity}}(z)$—remains constant at 0.6 HBs/w. Error bars for HBs/w values are $< \pm 0.05$ HBs/w.

Table S1: The effects of the degree of hydroxylation of the three studies silica surfaces on the HBs per water (HBs/w) formed between water and the surface, as well as on the intra-layer (adlayer) and inter-layer HBs.

Silica	HBs/w (surface-water)	HBs/w (Intra-layer)	HBs/w (Inter-layer)
3.5 SiOH/nm^2	0.2	1.0	1.6
4.5 SiOH/nm^2	0.3	1.7	0.9
9.6 SiOH/nm^2	0.5	2.3	0.6

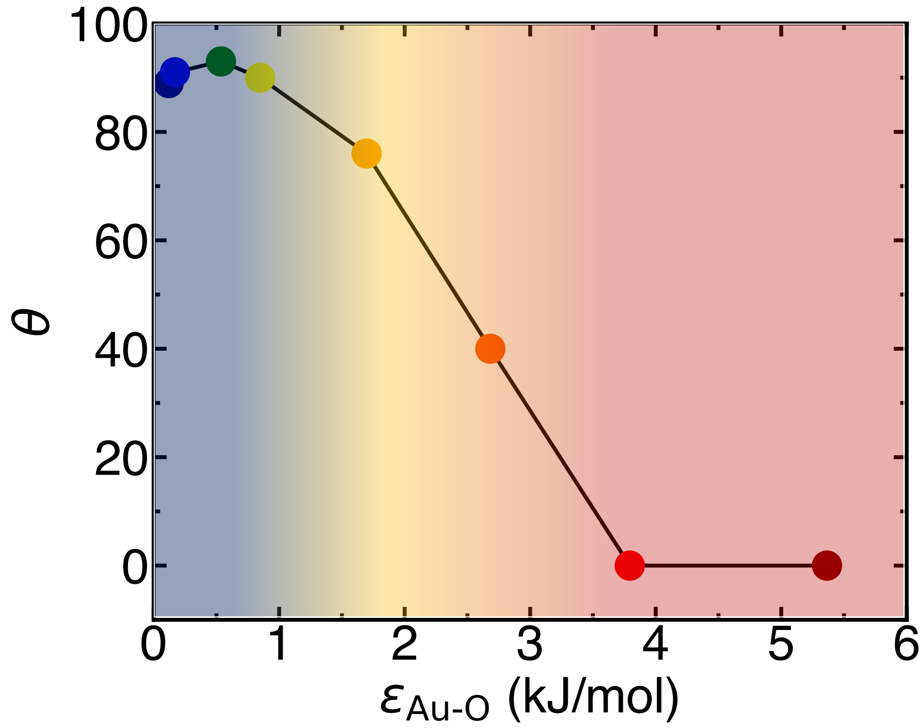

Figure S2: Contact angle θ (in degrees) as a function of surface–water interaction strengths (ϵ_{Au-O}, in kJ/mol), at z$_{im}$ = 1.0 Å, and $\Delta V = 0V$. At low interaction strength, large contact angle values indicate non-wetting behavior. As the interaction strength increases, the contact angle decreases and eventually reaches zero, signifying complete wetting.

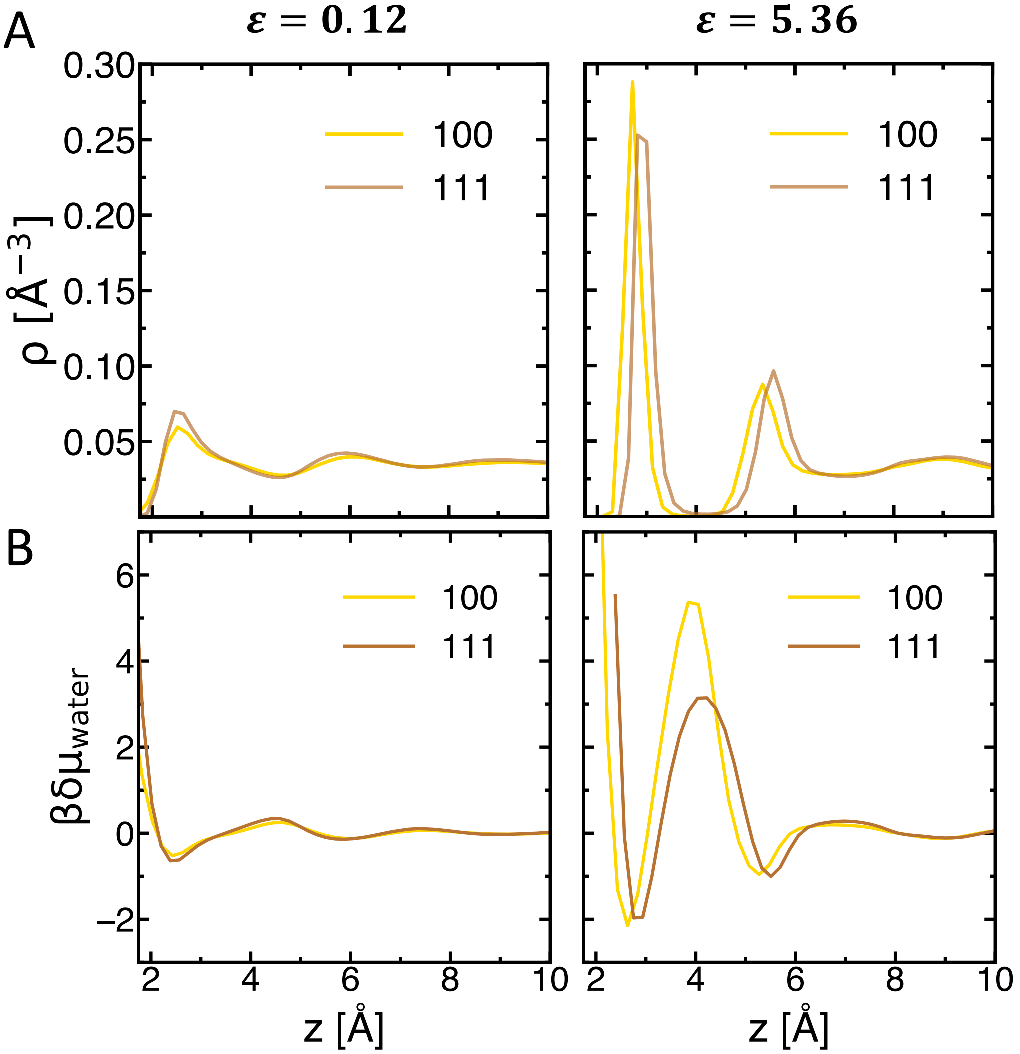

Figure S3: The effect of surface topology ((001) vs (111)) on interfacial water properties in the hydrophobic ($\epsilon_{Au-O} = 0.12$ kJ/mol) and amphiphilic ($\epsilon_{Au-O} = 5.36$ kJ/mol) domain: (A) Density profiles. (B) Water chemical potential $\delta\mu_{\text{water}}$ across the interface. Both hydrophobic and amphiphilic interfaces are studied at $z_{im} = 1.0$ Å, and $\Delta V = 0V$ conditions.

References

(1) Jo, S.; Kim, T.; Iyer, V. G.; Im, W. CHARMM-GUI: a web-based graphical user interface for CHARMM. *J. Comput. Chem.* **2008**, *29*, 1859–1865.

(2) Choi, Y. K.; Kern, N. R.; Kim, S.; Kanhaiya, K.; Afshar, Y.; Jeon, S. H.; Jo, S.; Brooks, B. R.; Lee, J.; Tadmor, E. B.; others CHARMM-GUI nanomaterial modeler for modeling and simulation of nanomaterial systems. *J. Chem. Theory Comput.* **2021**, *18*, 479–493.

(3) Berendsen, H. J. C.; Grigera, J. R.; Straatsma, T. P. The Missing Term in Effective Pair Potentials. *J. Phys. Chem.* **1987**, *91*, 6269–6271.

(4) Marin-Laflèche, A.; Haefele, M.; Scalfi, L.; Coretti, A.; Dufils, T.; Jeanmairet, G.; Reed, S. K.; Alessandra, S.; Berthin, R.; Bacon, C.; others MetalWalls: A classical molecular dynamics software dedicated to the simulation of electrochemical systems. *J. Open Source Softw.* **2020**, *5*, 2373.

(5) VandeVondele, J.; Krack, M.; Mohamed, F.; Parrinello, M.; Chassaing, T.; Hutter, J. Quickstep: Fast and Accurate Density Functional Calculations Using a Mixed Gaussian and Plane Waves Approach. *Comp. Phys. Comm.* **2005**, *167*, 103–128.

(6) Perdew, J. P.; Burke, K.; Ernzerhof, M. Generalized Gradient Approximation Made Simple. *Phys. Rev. Lett.* **1996**, *77*, 3865–3868.

(7) Grimme, S.; Ehrlich, S.; Goerigk, L. Effect of the Damping Function in Dispersion Corrected Density Functional Theory. *J. Comput. Chem.* **2011**, *32*, 1456–1465.

(8) Goedecker, S.; Teter, M.; Hutter, J. Separable Dual-Space Gaussian Pseudopotentials. *Phys. Rev. B* **1996**, *54*, 1703–1710.

(9) Bin Jassar, M.; Yao, Q.; Siro Brigiano, F.; Chen, W.; Pezzotti, S. Chemistry at Ox-

ide/Water Interfaces: The Role of Interfacial Water. *J. Phys. Chem. Lett.* **2024**, *15*, 11961–11968.

(10) Pezzotti, S.; Galimberti, D. R.; Gaigeot, M.-P. Deconvolution of BIL-SFG and DL-SFG spectroscopic signals reveals order/disorder of water at the elusive aqueous silica interface. *Phys. Chem. Chem. Phys.* **2019**, *21*, 22188–22202.

(11) Cyran, J. D.; Donovan, M. A.; Vollmer, D.; Brigiano, F. S.; Pezzotti, S.; Galimberti, D. R.; Gaigeot, M.-P.; Bonn, M.; Backus, E. H. G. Molecular hydrophobicity at a macroscopically hydrophilic surface. *Proc. Natl. Acad. Sci. U.S.A.* **2019**, *116*, 1520–1525.

(12) Tuladhar, A.; Dewan, S.; Pezzotti, S.; Siro Brigiano, F.; Creazzo, F.; Gaigeot, M.-P.; Borguet, E. Ions Tune Interfacial Water Structure and Modulate Hydrophobic Interactions at Silica Surfaces. *J. Am. Chem. Soc.* **2020**, *142*.

(13) Ugliengo, P.; Sodupe, M.; Musso, F.; Bush, I.; Orlando, R.; Dovesi, R. Realistic models of hydroxylated amorphous silica surfaces and MCM-41 mesoporous material simulated by large-scale periodic B3LYP calculations. *Adv. Mater.* **2008**, *20*, 4579–4583.

(14) Nosé, S. Molecular Physics : An International Journal at the Interface Between Chemistry and Physics. A Molecular Dynamics Study of Polarizable Water. *Mol. Phys.* **2006**, *52*, 255–268.

(15) Godawat, R.; Jamadagni, S. N.; Garde, S. Characterizing hydrophobicity of interfaces by using cavity formation, solute binding, and water correlations. *Proc. Natl. Acad. Sci.* **2009**, *106*, 15119–15124.
